# Development of an ADL-practice guideline: The next step towards guidance in essential nursing care activities

**DOI:** 10.1093/geroni/igab046.3380

**Published:** 2021-12-17

**Authors:** Svenja Cremer, Michel Bleijlevens, Silke Metzelthin, Janneke de Man-van devan Ginkel, Sandra Zwakhalen

**Affiliations:** 1 Maastricht University, Maastricht, Limburg, Netherlands; 2 UMC Utrecht, Utrecht, Utrecht, Netherlands; 3 Maastricht University, Maastrichjt, Limburg, Netherlands

## Abstract

Supporting and respecting care receivers in Activities of Daily Living (ADL) lies at the essence of nursing care, irrespective of diagnosis or healthcare setting. ADL-care is an intimate form of caring, and therefore close and personal to the care receiver, aiming to enhance their independence and comfort. Even though ADL-care is indispensable and highly valued by care receivers, the scientific foundation of ADL-care is weak. This leaves nursing professionals with insufficient guidance as to what constitutes quality ADL-care and what activities are necessary and effective. Therefore, we developed an ADL practice guideline according to the framework of the Dutch Institute for Health (AQUA-guideline) and AGREE II. The guideline was developed over three stages: (1) Determination of the target population and scope, (2) Analysis of problems leading to guiding questions and answering these based on literature search and consensus, (3) Testing and validation. A multidisciplinary working group determined the purpose, target group, and five clinical questions. We used literature search and consensus procedures to answer these questions in close collaboration with care receivers and professional care providers. This guideline provides guidance for nursing professionals to choose appropriate ADL-care options in five modules covering recommendations: (1) Involving care receivers in ADL-related care choices, (2) Identifying ADL-care needs, (3) Choosing effective interventions to enhance ADL-independence and comfort, (4) Supporting informal caregivers in ADL-care, and (5) Using ADL-care for early detection of health problems. These modules are considered leading for future developments in essential nursing care and will be evaluated in a pilot implementation.

